# An augmented reality game to support therapeutic education for children with diabetes

**DOI:** 10.1371/journal.pone.0184645

**Published:** 2017-09-28

**Authors:** Andrés-Marcelo Calle-Bustos, M.-Carmen Juan, Inmaculada García-García, Francisco Abad

**Affiliations:** 1 Instituto Universitario de Automática e Informática Industrial, Universitat Politècnica de València, Valencia, Spain; 2 Departamento de Sistemas Informáticos y Computación, Universitat Politècnica de València, Valencia, Spain; Swinburne University of Technology, AUSTRALIA

## Abstract

Therapeutic education in diabetes helps patients take responsibility for self-control of their disease, and providing technological support systems facilitates this education. In this paper, we present an augmented reality game to support therapeutic education for patients with diabetes. Our game helps children (aged 5–14 years) to learn carbohydrate (carb) content of different foods. The game shows virtual foods on a real dish. The number of carb choices corresponding to the visualized food is also shown (1 carb choice = 10 grams of carbs). A study to determine the effectiveness of the game in terms of learning and perceived satisfaction and usability was carried out. A total of seventy children with diabetes participated in the study. From the results, we observed that the initial knowledge about carb choices of the children who participated in the study was low (a mean of 2 on a scale from 0 to 9). This indicates that therapeutic education for patients with diabetes is needed. When the results for the pre-knowledge questionnaire and the post-knowledge questionnaire were compared, it was shown that the children learned about carb choices by playing our game. We used two post-knowledge questionnaires (one post-knowledge questionnaire that contained the same foods as the pre-knowledge questionnaire and a second post-knowledge questionnaire that contained foods that were different from the ones on the pre-knowledge questionnaire). There were no statistically significant differences between these two different post-knowledge questionnaires. Moreover, the knowledge acquired was independent of gender and age. We also evaluated usability and perceived satisfaction. The children were satisfied with the game and considered that the game offers a high degree of usability. This game could be a valuable therapeutic education tool for patients with diabetes.

## Introduction

Diabetes mellitus, also known as diabetes, is a chronic disease that is triggered when the body loses its ability to produce enough insulin or use it effectively. Insulin is a hormone that is secreted in the pancreas that allows the glucose of the food to pass to the body's cells, where it is stored or converted into energy. A deficiency in insulin causes excessively high levels of blood glucose (hyperglycemia), leading to the development of serious, long-term microvascular and macrovascular complications. There are three main types of diabetes: Type 1 diabetes, Type 2 diabetes and gestational diabetes mellitus. In type 1 diabetes, pancreatic cells produce insulin (beta cells). These cells destroy themselves by an autoimmune mechanism, and the body stops producing the insulin needed. Type 1 diabetes mainly develops during childhood and requires exogenous administration of insulin. In type 2 diabetes, the body can produce insulin, but either not enough is produced or the body does not respond to its effects (insulin resistance). It develops mainly in adulthood, although its prevalence during adolescence has been increasing. It requires strict control of food and exercise as well as oral anti-diabetic medication. Advanced stages may require insulin therapy. Gestational diabetes mellitus occurs when the body cannot produce or use enough of the insulin required for gestation.

According to the DAWN2^™^ study [1], the number of people with diabetes worldwide is estimated to be 382 million, which could increase to 592 million in 2035. In the DAWN2^™^ study, the opinions of more than 15,000 patients or their relatives were collected in 17 countries on four continents. Diabetes is a highly prevalent disease that is responsible for a significant economic burden on society in most countries. For example, a study carried out by Cefalu et al. [2] found that, in the US, the total cost of diabetes and prediabetes rose 48% over the 5-year period from 2007–2012, growing from $218 to $322 billion for both medical expenditures and reduced productivity [3, 4]. Cefalu et al.’s study [2] estimated that the average annual economic cost of diabetes for each American is approximately $1,000 [4]. When expressed as the average annual burden for each person diagnosed with diabetes, the costs have increased by 10%, rising from $9,975 in 2007 to $10,970 in 2012 [3, 4].

Therapeutic education in diabetes (TED) helps patients to take responsibility for self-control of their disease. Patients need support systems to facilitate TED. The World Health Organization (WHO) defines TED as an educational process that is integrated in the treatment, whereby the competencies (knowledge, skills and attitudes) and the necessary support for self-control of the disease are provided to people with diabetes and their families. The aim is for patients to acquire knowledge about their disease and the basis for treatment in order to be able to integrate them into their daily lives in order to be able to prevent, recognize, and act in situations of acute risk and to prevent cardiovascular risk factors [5]. This education is key for proper control of the disease [6]. According to Ferrer-García et al. [7] the minimum content required for adequate TED includes: 1) knowledge about eating habits and physical activity; 2) issues related to self-control (self-monitoring of blood glucose, self-measurement of blood pressure and weight, and foot self-examination); 3) a description of the process of the disease and treatment options. According to WHO, health professionals dedicated to TED should have appropriate and specific training.

One of the conclusions of the DAWN2^™^ study [1] is that the vast majority (81%) of people with diabetes who had attended education programs considered them to be helpful. However, only 49% were participating in any such programmes. For family members, the gap was even greater. Approximately three quarters of family members found diabetes education programmes helpful, but only 23% participated in any diabetes educational programs. According to the DAWN2^™^ study, more than 40% of people with diabetes have never participated in training programs about their disease. This percentage rises to 78% in the case of relatives. When health professionals responded to the same questions, 65% of them were in favor of increasing the training of patients and families, and 66% of them demanded more diabetes educators. A total of 85% of the healthcare professionals stated that the only training resources available for patients and their relatives were printed brochures. Only 47% referred to face-group activities and 45% to web pages. The DAWN2^™^ study concludes that, as a result of this data, communication between doctors and patients, regardless of resources can be greatly improved. That study also indicated that both patients and families and health professionals agree on their demand for more training to enhance self-control of patients with diabetes. Meanwhile, Elliott et al. [8] carried out a study to assess diabetes self-management and education among people living with type 2 diabetes in Oman. Their study involved 309 patients. One of their conclusions was that “many patients displayed dangerous diabetes self-management and education knowledge gaps”. Their findings suggested “a need for improving knowledge transfer to people living with diabetes”. In the same line, Schäfer et al. [9] carried out a study for analyzing patients' attitudes towards diabetes education. One of their conclusions stated that “physicians should encourage patients to participate in diabetes education”.

Proper control of nutrition is essential for the prevention of insulin resistance in order to slow its development and to prevent the occurrence of vascular and neurological complications due to poor glycemic control. For this reason, food is an essential part of educational programs for all patients with any form of diabetes. Nutritional recommendations focus on controlling daily intake in caloric terms, and its distribution in the number of meals as well as the distribution among proteins, fats, and carbohydrates. Carbohydrates have a direct relationship with the level of blood glucose. Therefore, knowing the number of carbohydrates that should be taken at each of the meals is essential for proper control of the disease. This control makes it possible to achieve a balanced diet and to maintain blood glucose levels that are close to normal.

On the other hand, there is clear evidence that interventions during the childhood and adolescence of diabetics have beneficial effects on glycemic control and psychosocial behavior, being even more effective in children than in adults [6]. Educational interventions for children and adolescents (and their families), must provide the basic and common principles of any quality education. They must be motivating and take into account where the learning takes place. They should be user-centric, usable, reliable, fun, entertaining, interesting, and understandable. The content must have smooth transitions (from easy to difficult) and be highly interactive, repetitive, and very practical. There must be clear objectives that solve real problems, and there must be reviews and summaries. The content must be evaluable by external review, and subsequent continuing education should be available. The interventions that have proven to be the most effective are those that can be integrated into the daily routine, those that adapt to the patient’s characteristics (age, etc.), those that focus on individualization for self-management by the patient, and those that make use of new technologies as a motivational tool [6]. All of these aspects were taken into account in the design of our game.

The use of games can improve learning outcomes [10] and they are a promising tool for motivating and engaging students [11]. Children are digital natives and enjoy everything that involves the use of technology. According to Glover [12], video games are especially appealing and motivating for children. Augmented Reality (AR) has also been used in games for educational purposes (e.g., [13]) and it has been demonstrated that AR promotes enhanced learning achievement [14]. Therefore, based on the enthusiasm of young for technology, the possibilities offered by AR, and the need of tools for TED, we present an AR game to support TED for children.

The first objective of this work was to develop an AR game to support TED for children. The second objective was to carry out a study of children with diabetes to determine the effectiveness of the game in terms of learning, perceived satisfaction, and usability. The first hypothesis is that children will increase their knowledge about carb choices of foods by playing our game. The second hypothesis is that there will not be statistically significant differences if the post-test questionnaire is the same as or different from the pre-test questionnaire.

The paper is organized as follows. Section 2 focuses on related previous work. Section 3 describes the game. Section 4 presents the description of the study. Section 5 presents the results, and Section 6 presents the discussion. Section 7 presents the conclusions and future research.

## Games, mobiles and augmented reality as educational tools

The terms gamification, game-based leaning or serious games are commonly used interchangeably to refer to the use of games or game elements in education. Gamification as a term was originated in the digital media industry in 2008. According to Deterding et al. [15], gamification refers to the use of design element characteristics for games in non-game contexts. Gamification in education has received increasing interest in recent years. For example, Matí-Parreño et al. [11] carried out a review in which they analyzed 139 papers published in journals indexed in JCR for the years 2010–2014. In that study, the authors identified the themes of research related to the use of games in education that had already been considered (effectiveness, acceptance, engagement and social interaction), among other aspects. They also suggested missing research themes such as personal drivers, emotions and design. They concluded that most of the papers were focused on the positive effects of gamification (e.g., increasing students’ motivation and interest in their learning process), and that negative effects remain unexplored.

The use of mobile devices for different purposes has been increasing. Specifically, the interest in mobile devices for education has been the target of several studies (e.g., [16, 17, 18]). Current mobile devices have a power that was unimaginable just 5 years ago. This is an optimal time for users/players to enjoy incredible experiences in the palm of their hands. There are two reasons for the success of mobile devices: the hardware that is available and the tools that are available for programming. Mobile devices not only include fast CPUs, large displays, cameras with 16 MP, graphics acceleration, compass/accelerometers, GPS sensors or gyroscopes, but they can also include depth and motion tracking sensors. The possibility to combine head-mount viewers with smartphones and have an AR platform has opened a new niche for research and the market, especially for education. Previous works have reported that mobile learning is highly motivating for students (e.g., [17]). Mobile devices can help in the learning process by creating more interactive and engaging environments [19]. Teachers can connect with their students on a more personal level with devices that the students use practically every day [20]. Mobile devices could facilitate versatility in the learning process since the learning activity could be performed at any place and time without requiring supervision [21]. Mobile apps can be personalized to each student [22]. Some reviews focused on specific aspects of mobile learning (e. g., [18, 23]). In the review carried out by Highfield & Goodwin [23], they analyzed the most popular Apps from the “education” section of the iTunes App Store at six-month intervals from April 2010 to October 2012. After classifying the Apps by the curriculum content, they observed that most of the Apps were identified as Literacy content (21%), followed by Science content (19%). They also identified less explored content (Economics and History), followed by Geography, Foreign Language, and Creative Arts.

With regard to games for diabetes education, several video games have been presented whose target audiences were children and adolescents (e. g., [24, 25]). Those games were designed to be played online (e.g., Captain Novolin, http://www.playretrogames.com/3283-captain-novolin), on consoles (e.g., Packy & Marlon [26]; Detective & Blocks Buildup [27]; Didget^®^ [28]), on computers (e.g., Egg Breeder [27], [24]), or on mobile phones (e.g., INSULOT [29]).

Android and iOS Apps for TED have also been presented. For example, mySugr (https://mysugr.com/) and Monster Manor (https://ayogo.com/blog/monster-manor, Kamel Boulos et al. [30]).

Most of the games for TED have a common bond, which is that they require players to balance food and insulin in order to keep the blood glucose character of the game within a normal range. Due to the proliferation of mobile devices, there are also numerous diabetes-related applications that can be downloaded (e.g., Play Store for Android and App Store for iOS). However, many of them are just informative and do not have any interaction. Others allow the storage of patients’ clinical data and generate graphs or reports, and even send this data to the specialist.

AR allows the inclusion of virtual elements in a real environment. A more precise definition is that AR combines reality and virtuality, allows real-time interaction, and has registration that is in 3D [31]. Current mobile devices can provide AR experiences in real time, which helps the users by adding information to the real scene to assist them in the tasks they are performing. Users can have the experience in any place and time. To achieve this, the device camera captures the real-world image and is overlapped by virtual elements. Even though AR has been used successfully in various fields (e.g., psychology [32]), it is not a technology that has been widely exploited in education. Nevertheless, AR for education has received increasing interest in recent years. Bacca et al. [33] conducted a review that includes 32 papers published in journals indexed in JCR between 2003 and 2013. One of their goals was to identify the fields of education in which AR was already being applied. The target fields were based on the International Standard Classification of Education [34]. Their analysis indicated that most of the studies (40.6%) were in the field of “Science”. Examples in this field are mathematics and geometry [35] or learning about the water cycle [13]. “Humanities & Arts” was the second field of education in which AR was applied the most (21.9%). Examples in this field are language learning [36] or learning about numismatics [37]. AR was applied in “Engineering, Manufacturing and Construction” (15.6%). AR was applied in “Social Sciences, Business and Law” (12.5%). AR was applied in “Services and Others (travel, transport, security services and hotel)” (6.3%) and in “Health and Welfare” (3.1%). An example in “Health and Welfare” is learning about dental morphology [38]. No studies have been presented in the fields of “Educational (teacher training at all levels of education)” or “Agriculture”. Another review was presented by Akçayir & Akçayir [14], in which they analyzed 68 papers about AR for education that were published in journals indexed in JCR. One of Akçayir & Akçayir’s [14] conclusions is that the most reported advantage of AR is that it promotes enhanced learning achievement.

To our knowledge, the only AR work that is related to diabetes was presented by Domhardt et al. [39]. In that work, the authors developed an AR system for mobile devices to estimate the carbohydrate portions (carb choices) of different real foods. Its main contribution is that the application is able to determine the volume of real food and infer its weight. The application uses a marker that is placed in front of the dish to be measured. The application shows a virtual mesh on real food. The user can interact with the virtual mesh to fit the exact volume. Their study involved 8 patients. According to Domhardt et al. [39], in 44% of the estimations, the error was reduced by at least 6 grams of carbohydrates. The participants identified several problems. Three of them were: “the drawing of the food was too complex; the precision of a measurement performed with the app was too imprecise; it was not possible to use the app for a dish of several mixed foods”. Similarly to Domhardt et al.’s work, our game uses AR for TED. However, the developments are totally different. From our point of view, AR realizes its full potential when virtual objects are 3D objects that look real and the user is interested in seeing these virtual objects from different points of view (360°). In other words, it is not the same seeing a 3D object that looks real than seeing a colored wireframe mesh. Moreover, seeing these virtual objects integrated with the real world offers added value. For example, it is not the same seeing an orange with a neutral background (e.g., blue) on the screen of a mobile device as seeing that orange on a real dish. When users are observing the augmented scene, they should feel that they are observing real food on a real dish. Our game includes these two features.

## Materials and methods

### Description of the game

**Fig 1:**
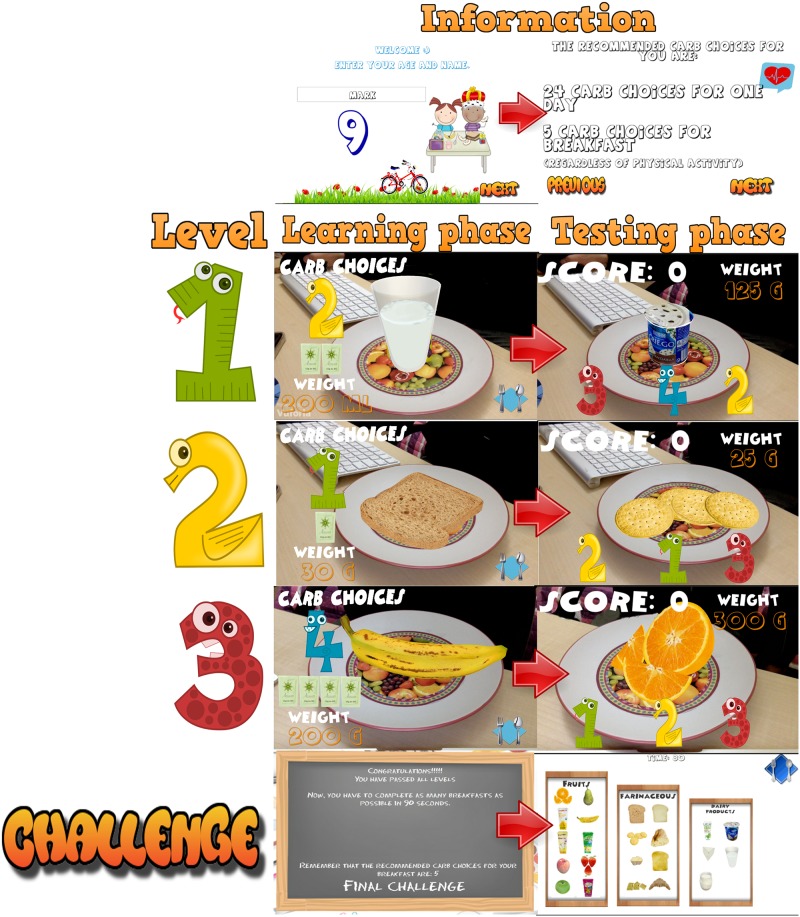
Scheme of the functionality of the game.

**Fig 2:**
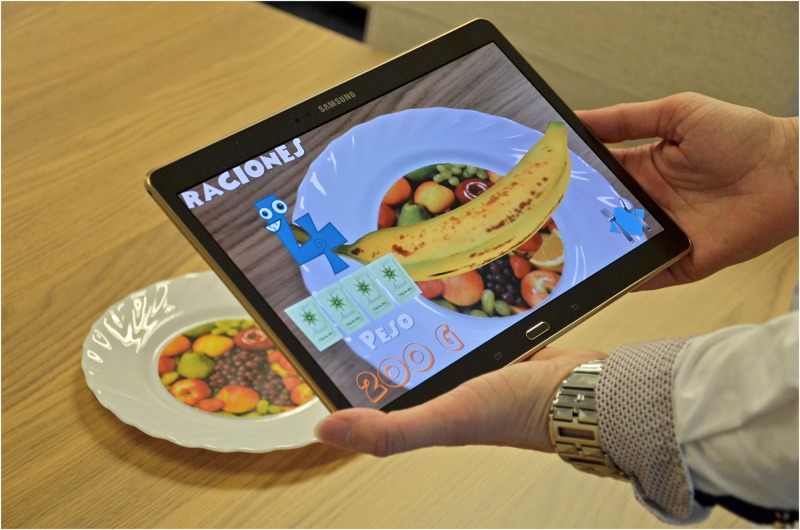
Example of fruits shown in the learning phase (A banana).

**Fig 3:**
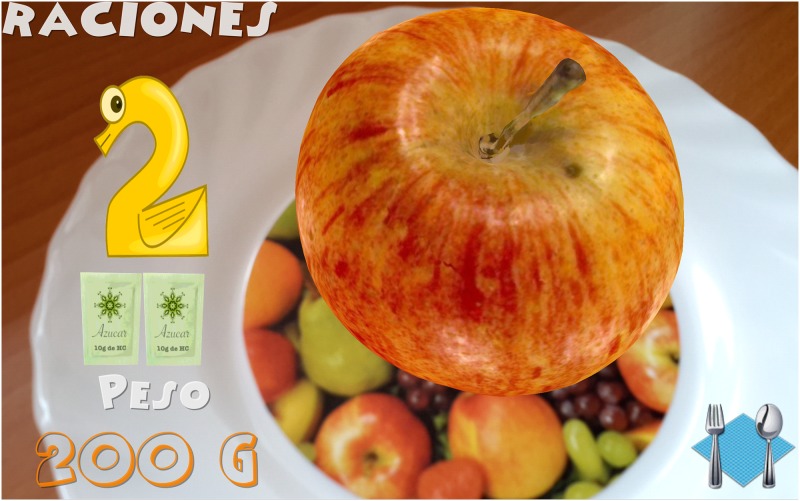
Example of fruits shown in the learning phase (An apple).

The game shows virtual food on a real dish. The goal is for the user to perceive food as if it were real food that was on a dish (without using any glasses). The game runs on a mobile device with an Android Operating System. When the game detects the target (an image in the center of the dish, e.g., Figs 1, 2 or 3), food appears in the center of the physical dish above the target. At this point, the user can zoom, rotate, raise, lower, or move the mobile device to observe the food from any position (360°). Another possibility is to move the physical dish in order to see the food from different positions and to zoom in or out. The steps in the game are as follows. Fig 1 shows a general scheme of the functionality of the game. A video demonstrating the functionality of our game can be found in the S1 File (a Supporting Information File).

**Fig 4:**
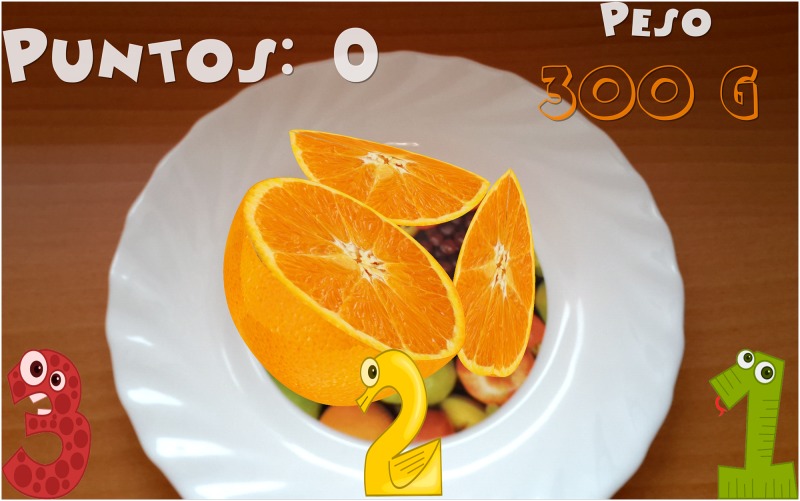
Example of an orange shown in the testing phase.

**Fig 5:**
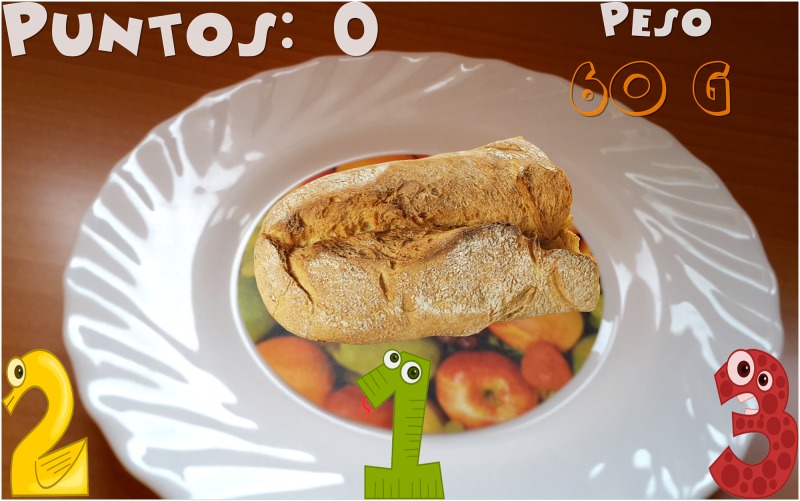
Example of a piece of bread shown in the testing phase.

**Fig 6:**
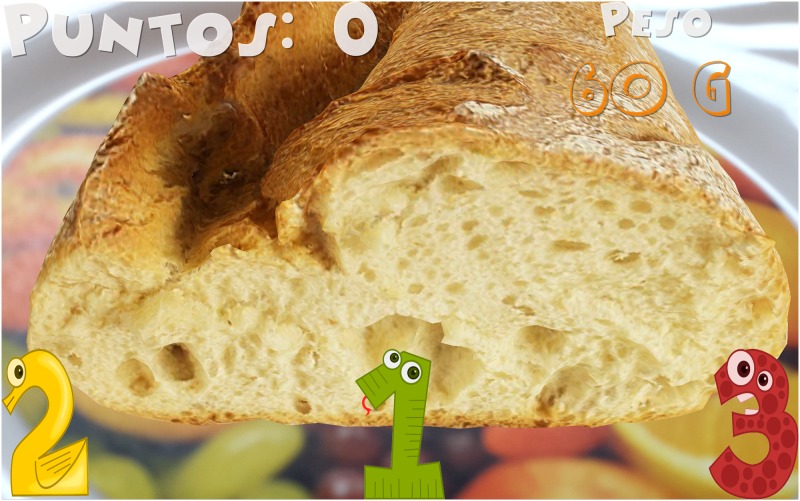
Example of a piece of bread seen close up in the testing phase.

**Fig 7:**
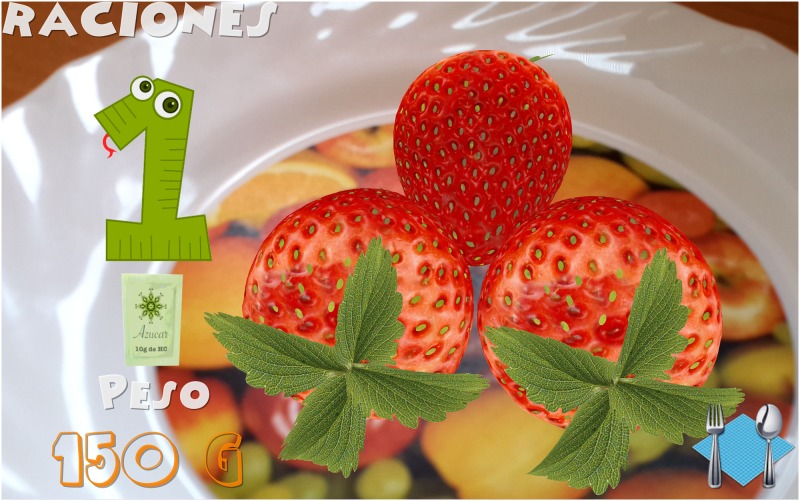
Example of three strawberries seen close up in the testing phase.

**Fig 8:**
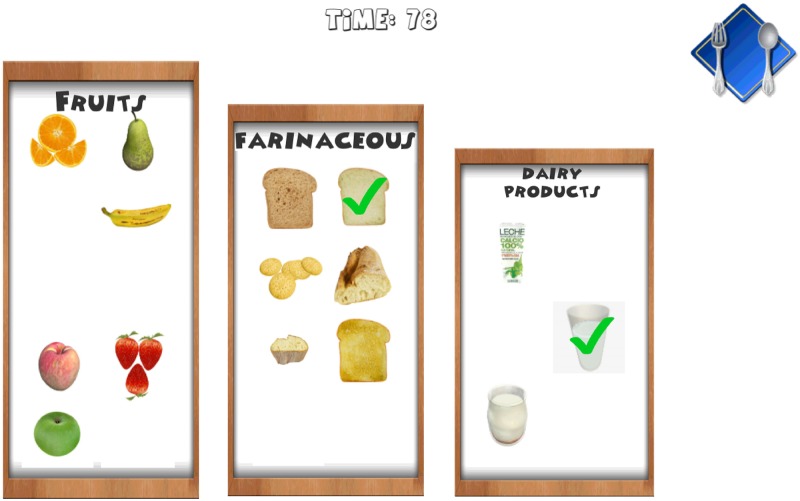
The final challenge.

1. Introduction of information. Initially, the child must indicate his/her age. After that, the game shows the carb choices recommended for his/her age for the whole day as well as the carb choices for breakfast (1 carb choice = 10 grams of carbs). This equivalence is currently being used in Spain and most of Europe. However, this equivalence is different in other countries. For example, in the US, Mexico, and most of Latin America, the equivalence is 1 carb choice = 15 grams of carbs. In Austria, the equivalence is 1 carb choice = 12 grams of carbohydrates.
2. The game. The game has 3 levels, each of which focuses on a food group. Specifically, the three food groups are: dairy products, farinaceous products (grains), and fruits. In the first level (dairy products), there are 6 foods. In the second level (grains), there are 8 foods, and in the third level (fruits), there are 10 foods. Each level has an initial learning phase in which foods are shown in real size along with their real weight and the number of carb choices are also shown. Figs 2 and 3 present two fruits that are shown in the learning phase of the third level. After this first learning step at each level, the user’s level of knowledge is checked (testing phase). In the testing phase, the user is asked about half of the foods used in the training phase. The food is shown in the center of the dish and the user has to choose the number that corresponds to the right carb choices among three possible options (numbers that appear in the lower area of the screen). Fig 4 shows an orange and Fig 5 shows a piece of bread in the testing phase. Fig 6 shows a piece of bread and Fig 7 shows three strawberries (see close up in the testing phase). The user must correctly guess 70% of all of the foods of each level in order to move to the next level; otherwise, the user returns to the training phase of that level.
3. The final challenge. After passing the three levels, a final challenge is proposed, which consists of identifying as many different breakfasts as possible in a given time (a minute and a half). The carb choices for each breakfast should be the ones recommended for the age of the child. To achieve this goal, the user has to choose foods from at least two different food groups. In Fig 8, the user chose a green apple, a slice of bread, and a glass of milk for a breakfast. The user has to press the button at the top right (with a fork and a spoon) to complete a breakfast.

### Design & development

For the design of the AR game, we counted on the advice of five experts in diabetes. The aspects that an educational intervention should have according to Lange et al. [6] were also considered for the design of our game. After knowing the problem and identifying the needs of this group, the functional specifications and the design of the game were defined.

The development of the AR game was performed using the defined design. For this development, we used Unity engine (also called Unity3D, http://unity3d.com) and Vuforia SDK.

The Vuforia SDK (https://www.vuforia.com) has an extension for Unity 3D, which facilitates the inclusion of animations and very complex virtual objects. These inclusions are not as easy if only Vuforia SDK is used. Vuforia uses computer vision techniques to recognize and track various types of fiducial elements in real time. In our case, we used the image that appears in Fig 2 as the Image Target. Unity and Vuforia make possible to develop applications for Android and iOS devices. We developed our own AR game for Android devices. However, once the application has been developed, the platform for which the application has been built can be selected and the same code can be used for building applications for Android or iOS devices. An overall flowchart of the game is available in the S2 File (a Supporting Information File). Extract of the C# code of the most relevant processes is also available in the S3 File (a Supporting Information File).

The development and testing of the AR game were achieved using a mobile device and a tablet with the Android operating system. The game is not device dependent and it can run on nearly any Android device. The recommended resolution for the camera is 5 MP or more. The only limitation is that, with a camera with low resolution (e.g., 2 MP), the application could have more problems recognizing the target. These problems can be solved with the appropriate illumination and the appropriate distance between the camera and the target.

The models used in the game were obtained mainly from two websites: www.blendswap.com and www.turbosquid.com. The models downloaded from Blend Swap were free, while the models downloaded from Turbo Squid required payment. After obtaining the models, it was necessary to perform modifications. We created several of the models. For the modification or creation of models, we used Blender (https://www.blender.org/), which is a free and open software for creating 3D models.

## Description of the study

This section presents the characteristics of the children that used the game, the measurements used during the study, and the steps followed.

### Participants

A total of 70 children participated in the study. There were 29 boys (41.4%) and 41 girls (58.6%). They were between five and fourteen years old. The mean age was 9.19 ± 2.38 years old. The total sample was divided into two groups. The children were randomly assigned to each group. In the first group (Group A), there were 24 boys (57.1%) and 18 girls (42.9%). In the second group (Group B), there were 17 boys (60.7%) and 11 girls (39.3%). These children were attending a conference for patients with diabetes and relatives in 2016.

### Measurements

**Fig 9:**
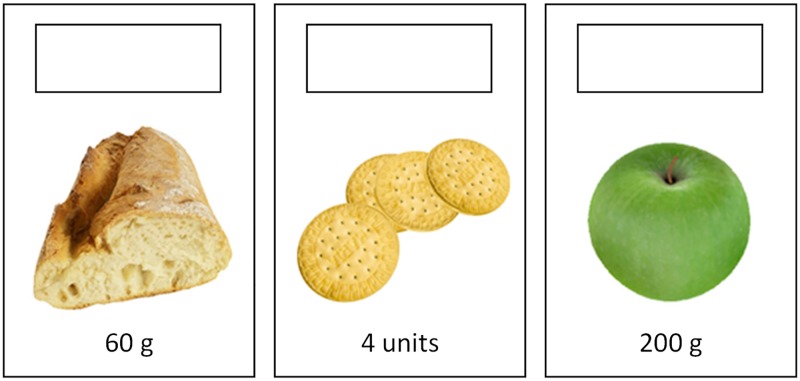
Example of three foods included in the knowledge questionnaire. The children must indicate the number of carb choices for the foods by filling out the upper box.

To retrieve the data for the analysis, three different questionnaires were used. There was a pre-test questionnaire (A) with the images of nine foods, in which the children should indicate the number of carb choices for the different foods. Fig 9 shows an example of three foods that were included in the knowledge questionnaire. This pre-test was designed to measure the knowledge the children had before using our game. Afterwards, the knowledge questions were asked again using two different questionnaires (B and C). All the foods that appear on the B and C questionnaires were included in our game. The B questionnaire had the same foods as the A questionnaire. The C questionnaire had different foods than the A questionnaire. Finally, the children filled out a satisfaction and usability questionnaire (D).

### Procedure

The study was conducted according to the principles stated in the Declaration of Helsinki. The University Ethics Committee of the Technical University of Valencia approved the research protocol and the written informed consent form that the parents signed. The data is available in the S4 File (a Supporting Information File). The procedure can be summarized as follows:

- Group A: Children that filled out a post-knowledge questionnaire (B) that was same as the pre-knowledge questionnaire (A).
- Group B: Children that filled out a post-knowledge questionnaire (C) that was different from the pre-knowledge questionnaire (A).

**Fig 10:**
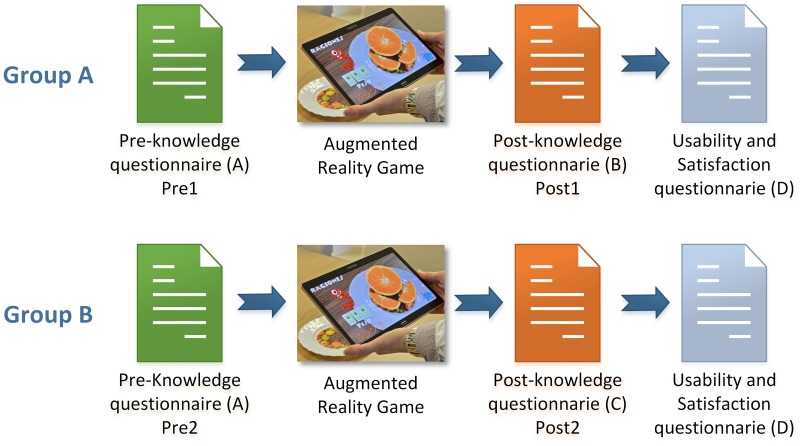
Study procedure.

Fig 10 shows graphically the procedure for both groups. The following protocol was used:

1. The children filled out the pre-knowledge questionnaire (A).
2. The children from both groups (Group A and Group B) played the game.
3. Then, the children from Group A filled out the post-knowledge questionnaire (B). The children from Group B filled out the post-knowledge questionnaire (C).
4. Finally, all of the children filled out the satisfaction and usability questionnaire (D).

## Results

This section presents the analysis of the data collected from our study. In order to explore means, standard deviations, and other measurements, an initial descriptive analysis was carried out. Then, data normality was checked, and the pertinent statistical tests were carried out based on those results. Before using inferential tests, Shapiro-Wilk (W = 0.909, p < 0.001**), Kolmogorov-Smirnov (D = 0.142, p = 0.007**), and Anderson-Darling (A = 3.507, p < 0.001**) tests were performed to check data normality. The three tests reported that our data did not fit the normal distribution. For this reason, the tests used were non-parametric (the Mann-Whitney *U* test for unpaired data, the Wilcoxon Signed-rank sum test for paired data, and the Kruskal-Wallis test instead of ANOVA). The data from the study was analyzed using the statistical open source toolkit R (http://www.r-project.org).

### Learning outcomes

In order to measure how much the children had learned, the knowledge variable was analyzed before playing (Pre-test) and after playing (Post-test). The knowledge variable was created by counting the number of correct answers for the nine foods. The statistical data are presented in the format (*median; interquartile range*), and all tests are presented in the format (*statistic U/W*, *normal approximation Z*, *p-value*, *r effect size*). ** indicates the statistical significance at level α = 0.05.

**Fig 11:**
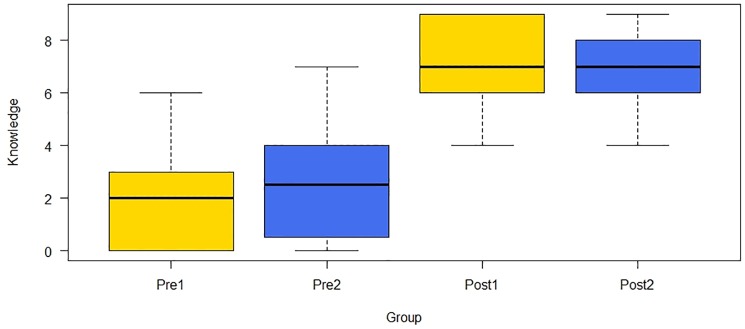
Scores of the knowledge variable before and after playing the game (the same post-knowledge questionnaire (Post1) vs. a different post-knowledge questionnaire (Post2)).

To determine whether or not there were differences between the initial knowledge of the two groups, an unpaired test was performed between the knowledge variable in Pre1 (1.95; 3) and the knowledge variable in Pre2 (2.5; 3.25) (U = 491, *Z* = -1.197, *p* = 0.234, *r* = 0.143). These results revealed that there were no statistically significant differences between the knowledge of the two groups in the pre-test. A paired test between Pre1 and Post1 (7.45; 3) showed that there were statistically significant differences between the scores that the children obtained before and after using the game and answering the same knowledge questionnaire (*W* = 0, *Z* = -5.661, *p* < 0.001**, *r* = 0.618). Another paired test revealed that there were also statistically significant differences between the scores of Pre2 and Post2 (6.86; 2) (*W* = 0, *Z* = -4.606, *p* < 0.001**, *r* = 0.616), which refers to before and after using the game and answering a different knowledge questionnaire. Finally, to determine whether or not there were differences between the two post-knowledge questionnaires, an unpaired test was performed between Post1 and Post2 (6.86; 2) (*U* = 723, *Z* = 1.662, *p* < 0.098, *r* = 0.199). These results showed that there was no statistically significant difference between the two post-knowledge questionnaires. Fig 11 shows the box plot for the scores for the two groups before and after playing the game.

The Kruskal Wallis test was also performed to take into consideration several other factors including Gender and Age. The results shown in Table 1 reveal that the knowledge acquired was independent from the Gender and the Age factors.

**Table 1:** The Kruskal-Wallis tests for the knowledge variable.

Factors	Kruskal-Wallis χ^2^	d.f.	*p*-value
Gender	0.202	1	0.654
Age	6.847	9	0.653

### Satisfaction and usability outcomes

The D questionnaire was used for the usability and satisfaction aspects. Table 2 shows this questionnaire, and the median and the interquartile range of the scores for each question. The answers used a Likert scale with values from 1 to 5. From the results of the questions, it can be observed that the median of perceived satisfaction and usability with our game was very high, with all the values being between 4 and 5.

**Table 2:** The results for satisfaction and usability. The possible answers for the questions were: 1. Strongly disagree; 2. Disagree; 3. Neither agree nor disagree; 4. Agree; 5. Strongly agree. The possible answers for question SA#7 were: 1. Never; 2. Hardly ever; 3. Sometimes; 4. Almost every day; 5. Every day. The possible answers for question SA#8 were: 1. Very bad; 2. Bad; 3. Regular; 4. Good; 5. Very good.

Id	Question	Median	Interquartile range	Answer
US#1	I found the game easy to use	4	2	Agree
US#2	I have gotten used to the game quickly	5	2	Strongly agree
US#3	I have concentrated more on playing than on the Tablet	5	2	Strongly agree
US#4	I could get close enough to the food	4	1	Agree
US#5	I could see the food from different positions	5	2	Strongly agree
SA#1	I have had a good time	5	0.25	Strongly agree
SA#2	I liked how the food looked on the dish	5	1	Strongly agree
SA#3	It seemed to me that the food on the dish could be real food	5	1	Strongly agree
SA#4	I think I have learned with this game	5	1	Strongly agree
SA#5	I would like to use these games to learn more about diabetes	5	1	Strongly agree
SA#6	I would invite my friends to use the game	4	1.25	Agree
SA#7	I would use this game again	5	1	Every day
SA#8	Score the game from 1 to 5	5	1	Very good

The global score variable combines all the answers of questions related to satisfaction and usability. To determine whether or not gender affected the global score, an unpaired Mann-Whitney *U* test was performed (U = 510.5, Z = -0.378, p = 0.710, r = 0.046). The results showed that there were no statistically significant differences for the global score between boys and girls. When the satisfaction variable (U = 447.5, Z = -1.199, p = 0.233, r = 0.146) and the usability variable (U = 482, Z = -0.749, p = 0.458, r = 0.092) are considered, there were no statistically significant differences for gender, in either case.

**Fig 12:**
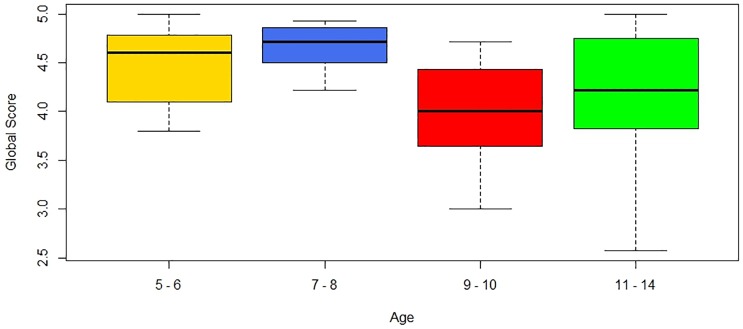
Boxplots of the global score variable for the four age groups.

A Kruskall-Wallis test was performed to determine whether or not age affected the global score (χ^2^ [3] = 11.821, p = 0.008**, r = 0.749986). We grouped the children into four age ranges (5–6, 7–8, 9–10 and 11–14). The results indicate that age affected the global score. The younger children gave higher scores. Fig 12 shows the box plot for the global score for the four age groups. When age is considered without grouping (χ^2^[9] = 18.841, p = 0.027**, r = -0.2942207), the conclusion is the same. When satisfaction variable for the four age groups is considered (χ^2^ [3] = 12.609, p = 0.0056**, r = 0.7734914), the younger children were more satisfied with the game. When age is considered without grouping, the same conclusion is obtained for the satisfaction variable (χ^2^[9] = 19.894, p = 0.019**, r = -0.2267709). With regard to the usability variable, when the four age ranges are considered (χ^2^[3] = 9.955, p = 0.019**, r = 0.659886), there were statistically significant differences in favour of the younger children. When the age variable is considered without grouping, the same conclusion is obtained for the usability variable (χ^2^[9] = 20.001, p = 0.018**, r = -0.3346588).

### Comparison of the augmented reality game with the ARSM task

To determine the usability and satisfaction of our game, we compared the scores for usability and satisfaction using the same questions as those used in the ARSM task [40]. The ARSM task is an augmented reality system that involves a user's movement to assess spatial short-term memory in healthy children. Similarly to our game, the ARSM task uses mobile Augmented Reality with children. A total of 76 children between 5 and 8 years old participated in the study of the ARSM task. The same age range was considered for the comparison carried out in this paper. When a global score variable that combines all of the answers of questions related to satisfaction and usability is considered in the two studies, there was no statistically significant difference between them (U = 893, Z = -0.544, p = 0.589, r = 0.054). Table 3 shows a comparison of our questions with the questions asked in the study of the ARSM task. When gender is considered for the global score, there were no statistically significant differences for the boys (U = 197.5, Z = -0.061, p = 0.961, r = 0.009) or the girls (U = 243.5, Z = -0.595, p = 0.559, r = 0.084) between the two studies. The children are grouped into two groups (the 5- to 6-year-old group and the 7- to 8-year-old group). A Mann-Whitney U test was applied. There were no statistically significant differences for the 5- to 6-year-old group (U = 166, Z = -1.864, p = 0.064, r = 0.259) or the 7- to 8-year-old group (U = 288.5, Z = 1.421, p = 0.159, r = 0.205) for the global score.

**Table 3:** Equivalence of the usability and satisfaction questionnaires between our study and the study of the ARSM task.

	Question identifiers					
**Our game**	SA#1	SA#2	SA#6	SA#7	SA#8	US#1
**ARSM Task**	SA#1	SA#2	SA#4	SA#5	SA#6	US#1

A Mann-Whitney U test was applied to the satisfaction variable in both studies (U = 1218.5, Z = 2.049, p = 0.041**, r = 0.205). There was a statistically significant difference in favour of our game. When gender is considered for satisfaction, there were no statistically significant differences for the boys (U = 239, Z = 0.965, p = 0.341, r = 0.136) or the girls (U = 365, Z = 1.961, p = 0.051, r = 0.277). When the 5- to 6-year-old group is considered, there was no statistically significant difference (U = 218, Z = -0.766, p = 0.450, r = 0.106). When the group of the 7- to 8-year-old group is considered, there was a statistically significant difference (U = 381, Z = 3.610, p < 0.001**, r = 0.521) in favour of our game.

A Mann-Whitney U test was applied to the usability variable in both studies (U = 988, Z = 0.215, p = 0.833, r = 0.021). There was no statistically significant difference between the two studies. When gender is considered for usability, there were no statistically significant differences for the boys (U = 188.5, Z = -0.300, p = 0.774, r = 0.042) or the girls (U = 309.5, Z = 0.809, p = 0.425, r = 0.114). When the 5- to 6-year-old group is considered, there was no statistically significant difference (U = 224, Z = -0.671, p = 0.510, r = 0.093). When the 7- to 8-year-old group is considered, there was no statistically significant difference (U = 279, Z = 1.242, p = 0.219, r = 0.179).

We analyzed the usability and satisfaction questions individually to determine whether or not there were statistical differences between our game and the ARSM task regarding age. There were no statistically significant differences for the 5- to 6-year-old group and for all the questions. There were statistically significant differences for the 7- to 8-year-old group and for the following questions (in all cases in favour of our game): SA#2 (U = 305.5, Z = 2.398, p = 0.017**, r = 0.346), SA#8 (U = 344.5, Z = 2.965, p = 0.003**, r = 0.428) and SA#9 (U = 346.5, Z = 3.091, p = 0.002**, r = 0.446).

## Discussion

In this paper, we present an innovative mobile AR game to support therapeutic education for patients with diabetes. The goal of our game is for children to learn about carb choices. Our game shows virtual food on a real dish and the patient has the perception of seeing a real dish with real food. Our work focusses on the positive effects of gamification, which is in line with current research [11]. Our game runs on mobile devices that the children are familiar with [20] so that initially they can engage with the game [19]. Moreover, considering that over a fourth of people in the world had access to a smart phone in 2016, a mobile game is the best option to reach as many people as possible. In this work, we supervised the learning process, but the game could be used at any place and time without our supervision, facilitating versatility in the learning process as has been point out in other works [21]. Several mobile AR systems for learning different topics have been presented in Section 2. However, to our knowledge, this is the first AR mobile game that helps patients learn about carb choices by combining a real dish and virtual foods. According to the review about AR trends in education carried out by Bacca et al. [33], one of the less explored fields is Health (3.1%), which is the field to which our AR game contributes.

The initial knowledge about carb choices of the children who participated in the study was low (a mean of 2 on a scale from 0 to 9). This indicates that therapeutic education for children with diabetes is needed. This result is in line with one of the conclusions of the DAWN2^™^ study [1], which indicated that patients, families, and health professionals agree on their demand for more training to enhance self-control of patients with diabetes.

With regard to learning about carb choices by playing our game, the statistically significant differences between the children’s knowledge before and after playing the game proved that games of this kind are suitable for transmitting knowledge. Therefore, our first hypothesis was corroborated. These results are in line with other studies that have demonstrated that, in general, gamification has good learning effects [41] and promotes interest in the learning process [11]. These results are also in line with other AR studies that have stated that the use of mobile AR for learning purposes can improve the learning process [13, 14, 21].

When the knowledge score after using the two different post-knowledge questionnaires was compared (one using the same elements as in the pre-test and the other using different elements from the pre-test), no statistically significant difference was found. Therefore, it can be concluded that the use of a post-questionnaire that uses the same or different foods than the initial questionnaire is not relevant. In any case, the children learned using our game. This indicates that the children were not only aware of recognizing the carb choices of the foods asked on the pre-test, but they also paid attention to all the foods shown in the game. Therefore, our second hypothesis was corroborated.

In this study, we did not compare the improvements in knowledge using our game with traditional methods or other types of methods (e.g., a video game with the same content, but without AR). In this regard, other works have compared the learning outcomes using AR systems and traditional methods. For example, Furió et al. [21, 42] obtained no statistically significant differences between AR systems and traditional methods for learning outcomes. Chiang et al. [43] compared two groups of students: an experimental group that used a mobile AR game and a control group that used a mobile game without AR. Their results showed that the average learning achievement of the experimental group was significantly better than that of the control group. Therefore, in previous works, an AR game compared to a game without AR or traditional methods offers greater or equal learning achievements. This observation suggests that the learning outcomes obtained using our AR game vs. non-AR game or traditional methods could offer greater or equal learning outcomes in favor of our AR game. However, a future study should be carried out to determine whether or not this hypothesis is corroborated.

Controlling eating is important for everyone in order to lead a healthy life; however, for a diabetic, self-control of food intake is a necessity [7]. The carb choices eaten at each meal are directly related to the insulin to be dosed. Therefore, knowing the number of carb choices contained in different foods would help patients to take responsibility for self-control of their disease and would improve their glycemic control. In this study, we did not focus on the improvements in the glycemic control of patients when using our game. However, this is a future work to consider.

To our knowledge, the work that is most closely related to ours is the work presented by Domhardt et al. [39]. As mentioned in the related work section, our proposal is totally different. However, it may be possible to join the ideas of these two works. In the work of Domhardt et al. [39], real dishes and real cooked foods are used. In our case, the dish is real and the uncooked food is virtual. A new version of our game could incorporate cooked foods.

With regard to usability, several authors have considered usability to be an important factor that affects educational effectiveness [44, 45, 46]. Sun et al. [47] argued that systems that are easy to use help students to focus their attention on the content. In our case, our game was easy to manipulate (with a mean above 4 on a scale of 1 to 5 in US#1). In addition, the people observing the children during the study stated that a great majority of children did not have any problems interacting with the game. Therefore, based on these considerations, our game does help students to focus their attention on carb choices. Moreover, we compared our data about usability with the data obtained in the study of the ARSM task [40]. There were no statistically significant differences between our game and the ARSM task. Both mobile AR systems offer a high degree of usability and for two different purposes.

With regard to satisfaction, there were no statistically significant differences between boys and girls, but the younger children were more satisfied with our game. We compared our data about satisfaction with the data obtained in the study of the ARSM task [40]. There was a statistically significant difference in favour of our game.

The children indicated that they would like to use these games to learn more about diabetes with a mean of 4.58 (on a scale of 1 to 5). This implies that the game would be well received as a therapeutic educational tool. In our opinion, the designed game can be an important educational resource because of its close relationship with the needs of children with diabetes and its highly motivational component as a tool to introduce or reinforce content about carb choices.

Finally, the current situation of AR in particular and ICT (Information and Communications Technology) in general and their presumable evolution suggest that the range of possibilities for the development of new tools for therapeutic education in diabetes is great. For example, Nao robots as pets have already been used to personalize health education for children with Type 1 diabetes [48].

## Conclusions

This work presents a mobile AR game to support therapeutic education for children with diabetes. From the results, we can affirm that the children acquired new knowledge about carb choices in the short-term by playing our game. Games of this kind facilitate versatility in the learning process since the learning activity could be performed at any place and time. The children only need a minimum setup with some images printed on paper and the handheld device. The features of our AR game and its minimal requirements position it as a pervasive educational game with great potential for therapeutic education in diabetes.

In this paper, we have studied whether the game can help in therapeutic education in diabetes. However, several comparisons are possible for future work (e.g., long-term learning). To do this, two groups (with or without training) could be considered. The game could be enhanced by adding more foods, and by adding other meals and not just breakfast. Also, other languages could be incorporated, and the number of grams equivalent to a carb choice could be adjusted based on the user's country. Validation in other countries could also be performed. We hope to benefit therapeutic education in diabetes with the work and ideas presented here.

## Supporting information

S1 File Video. Demostrative video of the game (Text in Spanish).
(MP4)

S2 File Flowchart. An overall flowchart of the game.
(PDF)

S3 File Pseudocode. Pseudocode of the most relevant processes.
(TXT)

S4 File Raw data file. Data used in the analysis.
(XLSX)
