# Supplementary figures and images for: An augmented reality game to support therapeutic education for children with diabetes
Source: PLoS One. 2017 Sep 28;12(9):e0184645. doi: 10.1371/journal.pone.0184645 (PMC5619721; doi:10.1371/journal.pone.0184645)

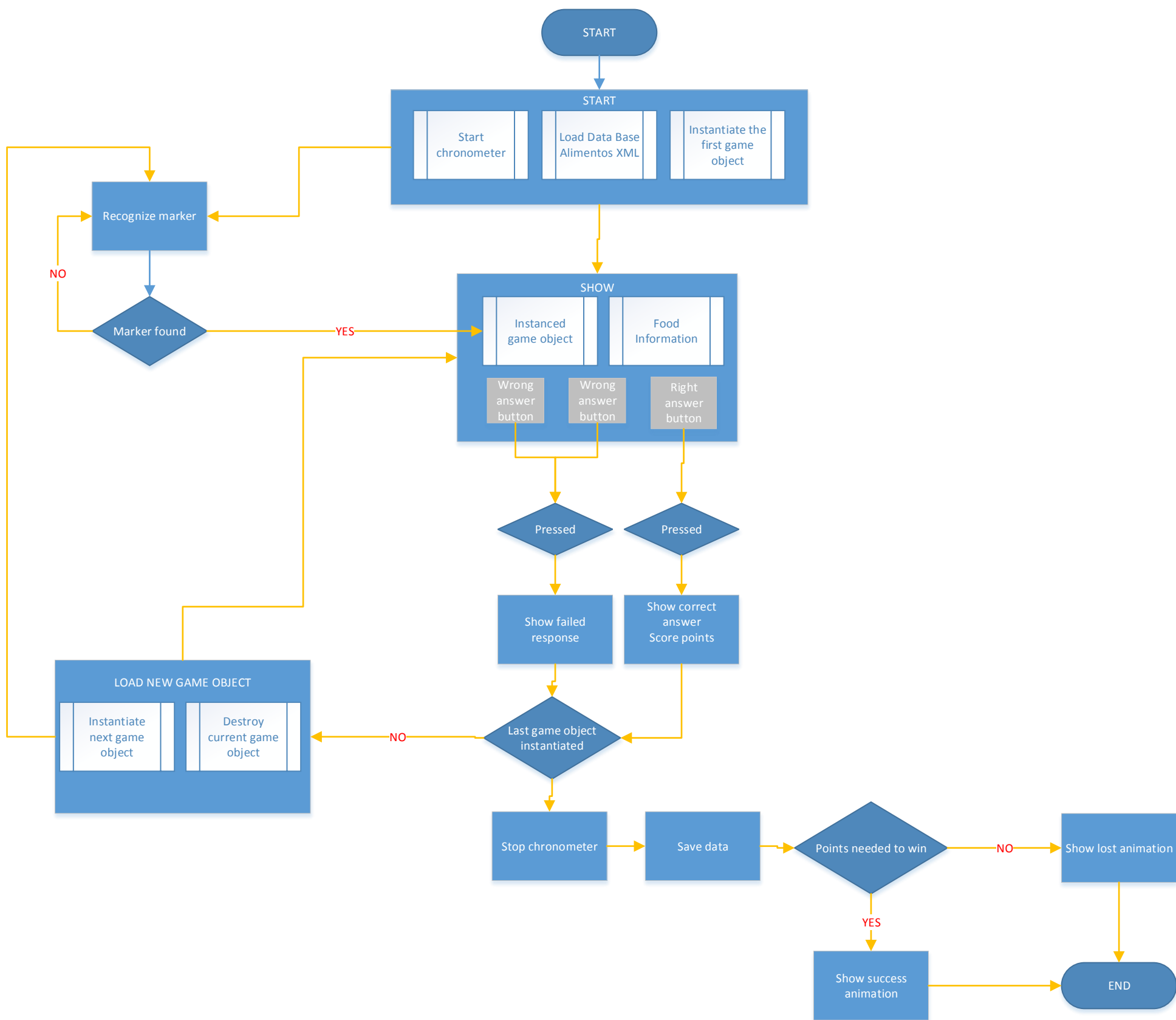

Supplement: S2 File — An overall flowchart of the game. (PDF) [file pone.0184645.s002.pdf]
